# Union Meeting of the Connecticut Valley Dental Society, the New England Dental Society, and the Connecticut State Dental Association, at Springfield, Mass., October 23, 24, and 25, 1889

**Published:** 1890-04

**Authors:** 


### UNION MEETING OF THE CONNECTICUT VALLEY DENTAL SOCIETY, THE NEW ENGLAND DENTAL SOCIETY, AND THE CONNECTICUT STATE DENTAL ASSOCIATION, AT SPRINGFIELD, MASS., OCTOBER 23, 24, AND 25, 1889.[1]

(Continued from page 167.)

### DISCUSSION OF "PRACTICAL POINTS IN DENTAL PATHOLOGY AND THERAPEUTICS."

*Dr. C. A. Brackett.*—In opening the discussion of this subject, it was my purpose to speak briefly of one or two classes of cases that are not the most satisfactory of those that come into our hands for treatment. Most of us have patients from the higher walks in life, patients of abundant means and of best dispositions in caring for their dental organs themselves. We also have patients that are the reverse of this; and then we have all grades of character between these two extremes. Probably very few of us escape having patients for whom sacrifices are made of organs that ought to have been attended to. The patients regret these sacrifices, and we regret them, and would gladly change the circumstances that decide these things, if we could. It may be of frequent occurrence for a patient to say, "I have teeth which I would like your advice on. They have given me a good

---

[1] Reported for the INTERNATIONAL DENTAL JOURNAL by Geo. A. Maxfield, D.D.S., Holyoke, Mass.

deal of trouble, but I would like to have them filled and saved." The patient is not abundantly blessed with means, or has not had time; and these are the difficulties of the situation. It seems to me that here is an opportunity to do some of the most profitable work. If not with that tooth, then with reference to the other teeth; and if not for that patient, then for the friends and family who will be influenced by that patient to come to you. We then can say to this patient to this effect: "These teeth have been neglected most sadly, permitted to ache, to ache repeatedly, to ache until you could no longer tolerate it, and you are forced to ask my assistance in saving them for you. It is most desirable for you to save these organs. I shall be happy to use my very best efforts possible to restore them to usefulness; but you should clearly understand these facts: that every hour's pain, of suffering endured with exposed pulp, in its present pathological state, which adds to the uncertainty of the results of my efforts, and to the lessened usefulness of the teeth to you in the future, has placed certain difficulties in the way. In order to get the teeth back to their normal condition you have got to retrace all the steps that have been taken. If they have been aching only a few hours, my work is comparatively easy; if at intervals of weeks or of months, my work is complicated, and it will consume a great deal of your time and be of greater trouble to me."

This can be explained to the comprehension of the intelligent patient who has the disposition to do the right thing; and is an opportunity for us to be very useful, to explain to patients the desirability of giving the dentist an opportunity to exercise his skill under the most favorable circumstances. The patients are not to blame for doing as they do. The judicious saving of the natural teeth is of the greatest importance. We do see instances in which the patient comes to us and asks to have a molar extracted; that is the only substantial bulwark they have in holding the jaws together correctly. We see the unfortunate disregard that has led to the extraction of all the molars on the upper and lower sides of the mouth. It seems a matter of the greatest importance.

Another class of unsatisfactory patients that comes to us consists of children with aching deciduous teeth, and on this subject I would be glad to be instructed myself. There is hardly a patient who comes in my office that I have more dissatisfaction with than children from poor, ignorant families who are suffering pain from exposed pulps. The child is suffering, and there is not the understanding, time, money, or disposition to make conservative operations. In some families, if the temporary teeth were cared for in the best possible way, it would absorb all the bread earnings of the family. In these cases I know no other way than to pursue the one that does the least harm. If I can keep the temporary tooth in its place, I like to do it. If I can make applications myself, I will do so. It may be a case that needs local depletion, or if an extreme case, it may need the application of caustics. If I could not do better, and feeling especially that the second set of teeth would have no more care than the first set was having, and that the molars would be sacrificed at an early age, as the easiest way out of difficulties,—the children need rest from pain and the parents need their sleep,—I should feel justified in extracting the tooth. It would be a matter of choice of evils. If there are other ways to get out of these difficulties, and if any others have suggestions to make, I shall be glad to learn about them.

*Dr. E. A. Stebbins.*—Is there any objection to putting arsenic in a temporary tooth?

*Dr. Brackett.*—I have not the fear of arsenic that many men have. If I were sure I was applying it to an exposed pulp and not to a perforation in the division of the roots, and have sealed it in to my satisfaction so as not to come in contact with the gum, I should have no fear whatever. In such cases as ordinarily exist, I should make the time of application as short as possible. I have no hesitancy whatever in using arsenical paste. We know that in nineteen out of twenty of these cases the pain is inside of the cavity in the temporary molar. Very likely the gum is growing into the cavity. The sealing of the destructive paste in these cavities must be done very carefully. I seal it with that which I think will make the sealing most secure,—gutta-percha or whatever else will do it. In regard to the pain, it depends on when the arsenic is applied; and the way in which that treatment is received will depend upon the state in which the pulp has been. In a pulp that has been exposed for months to all manner of irritation, no ordinary means of relieving pain will at all suffice. "There is no peace for the wicked" until destruction has been accomplished. All means will fail; you may keep your patient for hours, and yet the pain does not cease. In an instance of that kind you may apply arsenic and have everything comfortable quickly. That pulp is in a state of low vitality, and to my mind the arsenic just pushes it over. On the other hand, if you apply the arsenic to a recently exposed pulp in a large molar of a vigorous person, you get an entirely different result.

*Dr. Maxfield.*—What would be your treatment of temporary teeth after applying the arsenic?

*Dr. Brackett.*—That must depend on all the circumstances of which I have been speaking. Try to do the best under existing circumstances. In many cases, after removing the arsenic, that is all we can do for the teeth. The parents who would do nothing for the care of their own permanent teeth will do nothing for the children's temporary teeth. If you can make everything clean and wholesome, and fill them, by all means do it; and if you cannot, then do as well as you can. If we are to speak of the best thing to do for temporary teeth, in cases of this kind, it would be the removal of the destroyed pulp, and thoroughly to disinfect the canals and cavities. We may be justified in filling the cavity and providing an exit, which is an objectionable thing to do with permanent teeth. The circumstances may be such that we may think this is the best way out of the difficulty.

*Dr. N. Morgan.*—What do you fill the canals of temporary teeth with?

*Dr. Brackett.*—If I can clean them to my satisfaction, and have everything in a healthy state, I fill with a solution of gutta-percha in chloroform.

*Dr. Stebbins.*—Between what ages would you apply arsenic to the temporary teeth,—or does age make any difference?

*Dr. Brackett.*—It does make a difference. If the age approaches the time for these teeth to be lost, there is less occasion for using anything in the roots, whose absorption has already commenced.

*Dr. W. F. Andrews.*—I would like to know whether a tooth is more liable to pain if the arsenic is put in without uncovering the pulp or less liable by freely uncovering it?

*Dr. Brackett.*—The instance must be very rare where it is necessary to place arsenic in a tooth when there is no exposure. I should say it is best always to uncover the pulp.

*Dr. Stebbins.*—Did you ever see any difficulty in the development of the permanent bicuspid where arsenic has been placed in the temporary molar?

*Dr. Brackett.*—Never saw or heard of such an instance.

*Dr. Stebbins.*—When a person comes to you with a permanent tooth aching, what are the indications warranting you to devitalize the pulp?

*Dr. Brackett.*—I am guided first by the history of the case. The longer the tooth has been a source of offence, the less the chances are for successful capping. Besides the history of the case that the patient gives me is the appearance of the pulp as I investigate. A great failure in our treatment of exposed pulps has been in not discriminating as to its condition in the great range of the pathological state in which the exposed pulp may be. If the pulp were as large as the finger, and we could see the extent of the inflammation,—and ninety-nine out of every hundred commence in this pathological state,—we would not treat them so indiscriminately as we have in the past. There are individuals for whom you may cap an exposed pulp with about as much certainty of success as you fill an ordinary simple cavity. There are, we may say, governing circumstances that almost in themselves guarantee any pulp-capping, if the right conditions are observed. There are patients for whom any attempt at pulp-capping will be sure to result disastrously.

*Dr. A. H. Gilson.*—I am particularly interested at this time in what Dr. Brackett has said, as he has given us very good advice about how to treat these cases. I have a case which I would like to speak of. A patient recently came to me, and on examination I found a pulp which was very nearly dead. I treated this tooth to the best of my ability, and she came back the next day for me to finish filling the tooth, and in the mean time the tooth had not given her any trouble. When she left me I cautioned her not to eat any ice-cream or drink any ice-water; I afterwards found out that she went directly from my office and had a sherbet. On Sunday, which was two days after, she came out to my house, her face being badly swollen, and, as I had nothing at my house to treat teeth with, I tried to get her to go to a dentist up-town who would relieve her. She would not do this, but insisted on my going in to Boston, to my office, to attend to her. This I positively refused to do. She went off quite in a huff, and the next day I received a letter from a lawyer saying I was to be sued for malpractice, and I expect the sheriff at my house when I return to serve the writ.

To protect myself, I have had to place all my office fixtures, etc., out of my hands so as not to have a keeper placed there. This case will be tried before a jury, and if they defeat me, I can appeal to a higher court.

This is a question that concerns us all, as we are all liable to be tripped up in this manner. She says I caused the pain. What is there that we do that does not cause pain?

I would like to ask Dr. Brackett if he has noticed any difference in the development of the enamel of the bicuspids caused by the application of arsenic to a temporary tooth?

*Dr. Brackett.*—I, of course, shall admit that this may be possible. I have heard of half of the tongue being destroyed from the same cause; but I have never known of any instance of the kind.

*Dr. Stebbins.*—Will you give us your formula of arsenical paste?

*Dr. Brackett.*—I modify it in all manner of ways. Ordinarily, I use the preparation that White sells. Sometimes I use the clear arsenic, modifying it with the addition of acetate of morphia, oil of cloves, and various modifications that seem to me most likely to make the application with as little affliction to the patient as possible.

*Dr. S. S. Stowell.*—I would like to ask Dr. Brackett if he would make any distinction in applying the arsenic, whether the pulp was in a state of inflammation or in a state of rest? When it is in the state of inflammation, as we understand it, it is not capable then of absorbing the drug, and great pain is caused if applied at that time.

*Dr. Brackett.*—In any case where it is decided to destroy the pulp, I should first endeavor to relieve the pain; and to do this I would first treat with anodynes. If that failed, I would apply the arsenical paste. This condition, I apprehend, to be a condition of acute inflammation, accompanied with severe pain. In such a case as this, if I did not deem it advisable to apply the arsenic, I would try agents that would lessen the pain, to give opportunity for the inflammation to subside. If I deemed it best to apply the arsenic, and this being seriously painful, I would ask the patient to endure it only for a limited time. I would tell the patient to come back, if the pain was so severe he could not bear it, and I would take out the arsenic, and try the effect of an anodyne. An application for a limited time in this way will relieve the pain. It may not be successful immediately, but in a short time the pain will subside. So within an hour after you take out the arsenic your patient will be comparatively comfortable, and you can then replace the arsenic without having much more suffering.

*Dr. Miller.*—How long before the pulp will die?

*Dr. Brackett.*—I know of no therapeutic circumstance more uncertain than this. The pulp may be dead in a few hours and perhaps not in six months. I knew of an instance that came under the hands of one of Boston's best practitioners, for whom I have the highest respect, and one whom you all know. He tried most determinedly for two years before being successful in destroying a pulp. I have no hesitancy in leaving the arsenic in the tooth, provided it is properly sealed in. Sometimes we meet cases where a large portion of the pulp is dead, yet portions remain in the apex of the root that are a source of pain to the patient. This small fragment is a cause of dissatisfaction and discomfort so long as it exists. I would in such a case as this place the arsenic in the canals. I have, in teeth of a dense, hard structure, made applications for nine months before being able to destroy the pulp. It is a principle with me to carry the destructive agent against the tissue which it is intended to destroy.

*Dr. Stebbins.*—How long a time between the applications in the nine months?

*Dr. Brackett.*—I should be guided by the case. If a greater portion of the pulp has been destroyed with a great deal of difficulty and had extended over considerable time, and I have to make an application to destroy the remainder, after a week take out the dressing. If no progress has been made, try again; and after two or three weeks the progress has been slight, the next time leave it a longer interval. If my sealing is secure, I am satisfied to leave it for a long period. Another point is, the pulp may seem to have vitality left, as you first attempt to remove it, but often with a little persistence you can get it all away and avoid a second application.

(To be continued.)

